# Combinatorial MAB-Based Joint Channel and Spreading Factor Selection for LoRa Devices

**DOI:** 10.3390/s23156687

**Published:** 2023-07-26

**Authors:** Ikumi Urabe, Aohan Li, Minoru Fujisawa, Song-Ju Kim, Mikio Hasegawa

**Affiliations:** 1 Department of Electrical Engineering, Tokyo University of Science, Tokyo 125-8585, Japan; 4321510@ed.tus.ac.jp (I.U.); 4322542@ed.tus.ac.jp (M.F.); kim@sobin.org (S.-J.K.); hasegawa@ee.kagu.tus.ac.jp (M.H.); 2 Graduate School of Informatics and Engineering, The University of Electro-Communications, Tokyo 182-8585, Japan; 3 SOBIN Institute LLC, 3-38-7 Keyakizaka, Kawanishi 666-0145, Japan

**Keywords:** IoT, LoRa, lightweight distributed reinforcement learning, transmission parameter selection, multi-armed bandit problem

## Abstract

Long-Range (LoRa) devices have been deployed in many Internet of Things (IoT) applications due to their ability to communicate over long distances with low power consumption. The scalability and communication performance of the LoRa systems are highly dependent on the spreading factor (SF) and channel allocations. In particular, it is important to set the SF appropriately according to the distance between the LoRa device and the gateway since the signal reception sensitivity and bit rate depend on the used SF, which are in a trade-off relationship. In addition, considering the surge in the number of LoRa devices recently, the scalability of LoRa systems is also greatly affected by the channels that the LoRa devices use for communications. It was demonstrated that the lightweight decentralized learning-based joint channel and SF-selection methods can make appropriate decisions with low computational complexity and power consumption in our previous study. However, the effect of the location situation of the LoRa devices on the communication performance in a practical larger-scale LoRa system has not been studied. Hence, to clarify the effect of the location situation of the LoRa devices on the communication performance in LoRa systems, in this paper, we implemented and evaluated the learning-based joint channel and SF-selection methods in a practical LoRa system. In the learning-based methods, the channel and SF are decided only based on the ACKnowledge information. The learning methods evaluated in this paper were the Tug of War dynamics, Upper Confidence Bound 1, and ϵ-greedy algorithms. Moreover, to consider the relevance of the channel and SF, we propose a combinational multi-armed bandit-based joint channel and SF-selection method. Compared with the independent methods, the combinations of the channel and SF are set as arms. Conversely, the SF and channel are set as independent arms in the independent methods that are evaluated in our previous work. From the experimental results, we can see the following points. First, the combinatorial methods can achieve a higher frame success rate and fairness than the independent methods. In addition, the FSR can be improved by joint channel and SF selection compared to SF selection only. Moreover, the channel and SF selection dependents on the location situation to a great extent.

## 1. Introduction

The Low-Power Wide-Area Network (LPWAN) is a technology that enables low-power and long-distance communication for Internet of Things (IoT) applications [1]. The number of IoT devices using the communication protocols that belong to LPWAN has been rapidly increasing in recent years [2]. Among the LPWAN protocols, Long-Range (LoRa) systems attract attention because they do not require a license, but have an open standard. Besides, they can be built at a low cost. As a result, the number of LoRa devices is projected to grow to 730 million by 2023 [3]. Since the spectrum resource is limited, it may be difficult to support the communication of the massive number of increasing LoRa devices using traditional LoRa protocols. Hence, it is a critical issue to increase the number of LoRa devices in LoRa systems. To address this issue, it is necessary to adjust the transmission parameters of the LoRa devices, such as the Spreading Factor (SF), channel, transmission power, bandwidth, and distance, to adapt to the surrounding communication environment to maximize the spectrum efficiency. In this paper, we considered the selection of two main important transmission parameters that may affect the spectrum efficiency, i.e., the SF and channel. The impact of the transmission power, bandwidth, and distance on the communication capacity will be considered in our future work.

In LoRa systems, the Chirp Spread Spectrum (CSS) technique that uses a chirp signal whose frequency increases linearly with time is adopted, making the LoRa devices more resistant to interference [4]. The value of the SF determines the bit rate, the receiver sensitivity, and the Signal-to-Noise Ratio (SNR) threshold to correctly demodulate the signal. A smaller SF allows communication at a higher bit rate, but requires a higher SNR. Hence, communication is only feasible over relatively short distances and in paths with few obstacles for a smaller SF generally. Conversely, a larger SF is adapted to communications with longer distances but lower data rates. In addition, the orthogonality of different SFs can avoid interference among them [5,6]. Moreover, the theoretical Frame Success Rate (FSR) for Pure ALOHA, which is constantly employed in LoRa systems, also depends on the SF, the number of LoRa devices, and the duty cycle [7]. In Pure ALOHA, the LoRa devices access the channel randomly [8]. The LoRa systems constantly use the Pure ALOHA protocol without the concept of the time slots for communication, which makes them easy to implement. This simple protocol enables low-power communication and is suitable for IoT applications. For instance, assuming that there are 30 LoRa devices and each LoRa device sends 50 bytes of packets per 20 s in a setup similar to the experiments conducted in this paper, the theoretical packet transmission success probabilities for different SFs are different, which are as indicated in Table 1, along with the bit rate, receive sensitivity, and SNR threshold [9]. As described above, it is necessary to use an appropriate SF to transmit packets for the LoRa devices based on the distance between the GateWay (GW) to achieve higher communication performance and scalability in LoRa systems. In addition, the SF must be optimized according to the requirements of the LoRa applications in practical scenarios, such as the bit rate and the communication distance [10]. In addition to the SF, another communication parameter significantly impacting communication performance in LoRa systems is the channel [11]. The Pure ALOHA protocol does not perform carrier sensing, i.e., it does not sense the communication channel before sending packets, but communicates by random access. Furthermore, many devices, including LoRa devices, communicate using the Industrial, Scientific, and Medical (ISM) band since it is an unlicensed band. Hence, collisions and interference are prevalent in the communication link between LoRa devices and GWs in the IoT networks with massive numbers of IoT devices [12]. As the data traffic and load increase within the ISM band, network performance will be significantly degraded without proper channel selection, which significantly impacts the scalability of the LoRa systems. In addition, since packet collisions in LoRa systems occur when two or more LoRa devices transmit packets using the same SF and channel simultaneously, these parameters must be selected jointly and appropriately [13,14].

Research on communication parameter management in LoRa systems can be divided into centralized and distributed approaches. Most of the existing research focuses on centralized approaches in which the network server allocates communication parameters to the LoRa devices [15]. The GW is responsible for transmitting the LoRa packets of nodes and forwarding them to the network server [16]. The network server may allocate optimal transmission parameters for the centralized approaches. However, the GW needs to know much a priori information, such as the distance between the GW and the LoRa device, the packet length, the event probability, the number of devices, and so on, to determine the communication parameters for the LoRa devices in the centralized approaches, which may increase the communication latency. Furthermore, the LoRa device needs to be awake to receive the transmission parameters instruction from the GW, which may increase the energy consumption of the IoT devices compared to decentralized parameter selection methods. Moreover, the centralized approaches also increase the consumption of communication resources due to the transmission of the transmission parameters’ instruction. There are also some studies on improving the performance based on the standardized protocol of the LoRa systems. There are mainly three specifications for the LoRa systems, i.e., Class A, Class B, and Class C. Class A uses a so-called Pure-ALOHA-type asynchronous multiple-access scheme in which a terminal uplink has a short burst signal at an arbitrary timing. On the other hand, terminal reception is limited to a very short period immediately after the uplink. In Class B, all GWs and terminals use beacons transmitted by the GWs to synchronize with the network. By accurately recognizing the time at which each terminal opens its reception window, the GW can immediately send a call when there is downlink information. In Class C, high-speed downlink communication is possible because terminals can always receive signals. Even though the parameter control methods for different LoRa classes are not the same, the standardized protocols face the same issues as the other centralized methods. As described above, considering the future proliferation of LoRa devices and the need to provide ultra-long battery life for LoRa devices, only the resource allocation schemes that can significantly reduce signaling to the access network are feasible. Therefore, a decentralized approach is required where each LoRa device autonomously selects appropriate communication parameters without the help of the GW/network server [17]. Compared to the centralized approaches, the decentralized approach allows parameter selection without needing prior information and the transmission of the transmission parameters’ instruction [18]. Hence, the spectrum resource for the communications and energy consumption of the LoRa devices can be reduced.

Several decentralized communication-parameter-selection methods based on the Multi-Armed Bandit (MAB) algorithm have been proposed in previous studies to improve the scalability of the LoRa systems. However, these previous studies were limited to the selection of only the SF or only the CH. Meanwhile, few papers have considered the implementation of the methods in practice. As IoT devices have low computational power, limited storage, and less battery, it is a great challenge to develop a joint SF and CH method for practical LoRa systems. To address this issue described above, we proposed a MAB-based joint channel and SF-selection method in our previous work. We evaluated the performance of the proposed method in high-density static and dynamic practical environments [19]. The experimental results demonstrated that the performance of the FSR can be improved by selecting both the channel and SF. However, the selection of the SF and channel may be correlative, which was not considered in our previous work. In addition, the communication performance of the LoRa device strongly depends on the selection of the SF related to its location, which was also not considered. To consider the correlation of the SF and channel to improve the performance in our LoRa systems further, we set the SF–channel-selection problem as a combinatorial MAB-based SF–channel-selection problem and solved it using the MAB methods in this paper. Moreover, we evaluated the performance of our LoRa systems in the FSR with varied locations of the LoRa devices to show the relationship between the SF selection and the location of the LoRa devices. We consider that the proposed method may be a potential transmission-selection solution for the LoRa systems in the future. The main contributions of this paper are as follows:

- We set the SF–channel-selection problem as a combinatorial MAB-based SF–channel problem and introduced the MAB algorithms, including the Tug of War dynamics (ToW), Upper Confidence Bound 1 (UCB1), and ϵ-greedy algorithms to solve the formulated problem. In the MAB-based SF–channel-selection methods, the SF and channel were selected only using the ACK information by the LoRa devices, which can be applied without modifications to the LoRa protocol. In addition, since the operation of the MAB algorithms is not complex, the methods can be easily implemented in IoT devices with memory and computational power constraints.
- We evaluated the proposed method in experiments with real-world LoRa devices in an environment where the LoRa devices were distributed in multiple indoor locations. First, we evaluated the performance of the FSR and the relationship between the selection rate of the SF and the locations of the LoRa devices when only selecting the SF. The results demonstrated that the appropriate SF depended on the distance from the GW. Besides, the superiority of the MAB-based SF-selection methods was demonstrated by comparing the methods with random access. Then, we evaluated the performance of the FSR and Fairness Index (FI) when considering a joint selection of the SF and channel. Specifically, to show the effectiveness of the proposed combinatorial MAB-based SF–channel method for the FSR and FI, we compared it with the independent MAB-based SF–channel method, where the SF and channel are selected independently. Next, we focused on the performance evaluation of the proposed MAB-based SF–channel-selection method. The performance of the FSR with varying numbers of LoRa devices, transmission intervals, and the locations of the LoRa devices was evaluated exhaustively.

The remainder of this paper is organized as follows. Section 2 provides an introduction to the related work. Section 3 describes the system model and the formulated problem. Section 4 describes the combinational MAB-based SF–channel-selection methods. Section 5 describes the implementation and performance evaluation of the proposed combinational MAB-based methods. Finally, we provide a conclusion to summarize this paper in Section 6.

## 2. Related Work

In this section, we describe the related work on communication-parameter-management-techniques in LoRa systems. We first present the SF- and channel-selection methods in the centralized approach, followed by the decentralized approach.

### 2.1. Centralized Approaches

In this subsection, we introduce the related work on the centralized approaches for SF allocation, channel allocation, followed by SF and channel allocation.In the centralized approaches, the transmission parameters are allocated by the GW/network server.

#### 2.1.1. SF Allocation Methods

Simple centralized methods for allocating SFs include the Equal-Interval-Based (EIB) and Equal-Area-Based (EAB) allocation schemes [15,20]. In these schemes, the total network area is first divided into concentric circles, assuming that the GW is at the center of the area. The EIB then divides the network to make the width of each annulus equal, while the EAB divides the network to make the area of each annulus equal. Next, the SF is allocated according to the proximity to the GW for the annuli. The smaller SF is assigned to the annuli that are near the GW. In [15], the EIB and EAB methods were analyzed and compared. These simple schemes are based on the idea that the reception strength weakens with the increase of the distance from the GW. However, in a real network environment, it is necessary to consider the effects of interference and fading in a particular region, as well as the channel conditions. Therefore, these methods are challenging to improve the scalability sufficiently in real-world environments. To this end, approaches to parameter optimization were proposed in [21,22,23,24], where the problem for resource allocation was formulated as the optimization problem, and optimization solvers were proposed to solve the formulated problems. Some other methods that allocate the SF based on channel gain and the Signal-to-Noise Ratio (SNR) were proposed in [25,26], respectively. In [27], a modification of the existing LoRa@FIIT protocol was proposed, ensuring energy-efficient, QoS-supporting, and reliable communication over the LoRa technology by selecting an appropriate SF and transmission power. In [28], a deep-reinforcement-learning-based adaptive PHY layer transmission-parameter-selection algorithm was proposed to select the SF and power. The proposed algorithm was run on the GW to allocate the SF and power for the LoRa devices. It was shown that the proposed algorithm could achieve 500% packet delivery ratios in some cases while being adaptive at the same time. However, these centralized approaches for SF selection require the GW/network serverto know a priori information, such as the number of devices, their locations, and transmission probabilities. Furthermore, the GW/network servermust send control signals regarding the communication parameters to all LoRa devices, which leads to increased communication resource consumption and communication latency.

#### 2.1.2. Channel Allocation Methods

In addition to the SF, as discussed in the previous section, the management of the channel also has a significant impact on the scalability in the LoRa systems. The quality varies greatly from channel to channel in the unlicensed ISM band because it is susceptible to interference from IoT devices and electronic devices in other applications. Similar to the SF-selection methods, many methods have been proposed [18,29]. However, these existing studies have disadvantages, such as the need for GW/network serverto know prior information, as well as the centralized approach in the SF-selection methods, which increases the communication resource consumption since the transmission parameters instructionneed to be sent from the GW/network server.Because the disadvantages described above will become more serious with the future increase of the number of IoT devices, most of the related centralized approaches would have difficulty becoming realistic solutions.

#### 2.1.3. SF and Channel Allocation Methods

Reference [30] proposed a joint channel- and SF-selectionmethod that allocates a SF-selectionvalue depending on the rate demand of each end-device and considers the availability of the frequency channel for each uplink transmission. However, the details of the proposed method were not fully given. According to the existing description, it seems that the transmission parameters could not be adjusted corresponding to the dynamic environments, while the correlation of the location of the users and SF selection, as well as the implementation of the method were not well considered. Moreover, it seems that the proposed method in [30] is a centralized method, which may face the same disadvantages as other centralized methods.

### 2.2. Distributed Approaches

In the decentralized approaches, it is possible to reduce communication resource consumption, latency, and energy consumption compared to the centralized approaches, since LoRa devices make decisions independently. However, most existing studies on parameter selection methods for the LoRa systems are centralized approaches, and there are limited related studies on distributed approaches. In this subsection, we describe the SF and CH distributed selection approaches.

#### 2.2.1. SF Selection Methods

SF-selection methods based on the MAB algorithm were proposed in [31,32]. In [31], SF selection based on a popular algorithm called Exponential Weights for Exploration and Exploitation (EXP3) was proposed and evaluated by simulation. In [32], a SF-selection method based on the Upper Confidence Bound (UCB), a MAB algorithm that can perform high-precision search, was proposed. The simulation results showed that the proposed method based on the MAB algorithm improved the success rate of data transmission. However, in the existing studies, SF-selection methods were proposed assuming that all LoRa devices use the same channel, which is unrealistic. In addition, the method has yet to be validated through real-world experiments, and realistic environments were not considered.

#### 2.2.2. Channel Selection Methods

Several channel selection distributed approaches were studied in [33,34]. In these approaches, the channels were selected based on the MAB methods. In [33,34], a channel-selection method using the UCB algorithm, a typical MAB algorithm, was proposed and implemented on an actual LoRa device. Moreover, the experimental results under a dynamic environment with changing channel states were presented. However, these studies assumed that all LoRa devices use the same SF, which is not a realistic assumption. Furthermore, only experiments with a small number of LoRa devices were conducted, and no investigations were conducted in environments with a large number of LoRa devices.

#### 2.2.3. SF and Channel Selection Methods

In [19], a method for simultaneous channel and SF selection was proposed for multiple MAB algorithms. However, only the performance under high-density conditions was evaluated. The distance between the LoRa devices and the GW and the reception strength have yet to be considered. Furthermore, the MAB problem structure considering the relevance between the channel and SF when selecting them simultaneously has yet to be well evaluated.

In summary, the existing studies on centralized methods have disadvantages, such as the need for the GWs/networks to know prior information and to send transmission parameters instruction to IoT devices, which increases communication resource consumption. Because the disadvantages described above will become more serious with the future increase of the number of IoT devices, most of the related centralized approaches would have difficulty becoming realistic solutions. Although studies on decentralized methods can solve the disadvantages of centralized methods, several issues have not yet been considered in the existing studies. For instance, the distance between the LoRa devices and the GW and the reception strength have yet to be considered. Furthermore, the MAB problem structure considering the relevance between the channel and SF when selecting them simultaneously has yet to be well evaluated. To solve these issues, we propose a combinatorial MAB-based joint channel- and SF-selection method in this paper, which will be exhaustively described in Section 4. The comparison of the relevant schemes is summarized in Table 2. ✓ and ✕ in the Table mean whether the reference considered the corresponding items or not.

## 3. System Model and Problem Formulation

**Figure 1:**
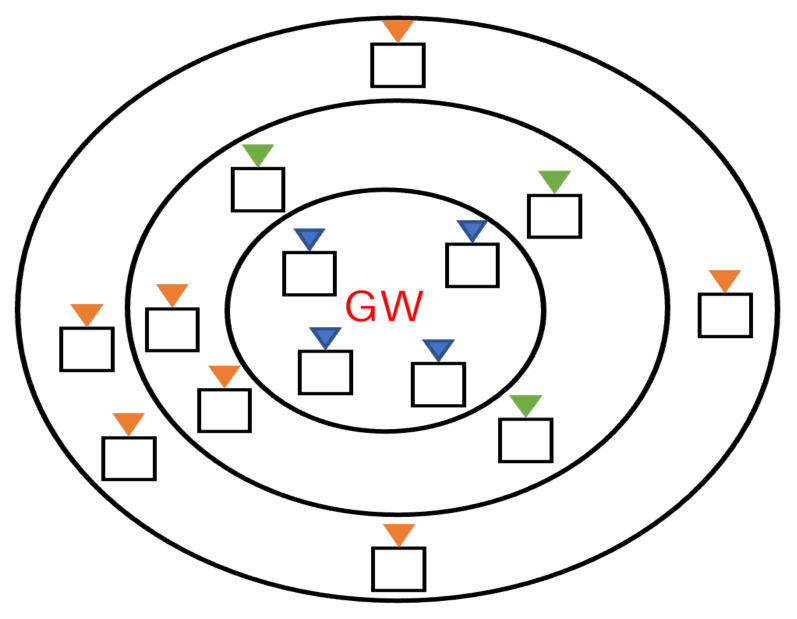
The distribution of SF and LoRa devices.

This paper considered the uplink transmission of a LoRa system with a star topology consisting of one GW and *L* LoRa devices. Denote $D=\{D_{1},D_{2},\ldots D_{l},\ldots ,D_{L}\}$ as the LoRa device set, where $D_{l}$ denotes the *l*-th LoRa device. Assume that the number of available channels for the LoRa devices is *I*. The public ISM band of Japan was used for the communications between LoRa devices and the GW in this paper, where the bandwidth of each channel was 125 kHz, while at most 15 channels can be used for communication. We considered a natural wireless communication environment where LoRa devices are distributed in various locations with different distances from the GW, as shown in Figure 1. In Figure 1, the concentric circles are divided according to the distance from the GW. Different colors represent different SFs assigned to LoRa devices, which may need to be assigned according to the distance between the LoRa device and the GW. Assume that the number of SFs is *S*. Each LoRa device selects one SF and one channel to transmit packets each time. As described in Section 1, LoRa employs CSS modulation so that signals with different SFs (7-12) can be identified and successfully received even if they are transmitted simultaneously on the same channel. In addition, different SFs have different transmission speeds and thresholds for the SNR that can be successfully received. Therefore, each LoRa device must select an appropriate SF considering its distance to the GW and interference effects in the surrounding environment. Theoretically, the spreading codes for different SFs are orthogonal, so collisions only occur when two or more LoRa devices choose the same SF and channel. In practice, however, perfect orthogonality may not be guaranteed, and the interference between transmissions using different SFs on the same channel must be considered [15,19]. In addition to the channel and SF, the bandwidth *B* and the transmit power TP also can be selected to improve the communication performance. The bandwidth can be chosen as 62.5 kHz, 125 kHz, 250 kHz, and 500 kHz. The transmit power can be selected from −1 dBm to 13 dBm, depending on the application requirements and the communication environment of the LoRa devices. In this paper, the bandwidth of the channel and the transmission power for all LoRa devices were set to 125 kHz and the maximum transmit power, i.e., 13 dBm, respectively. We assumed that all LoRa devices transmit *M*-byte packets with the same length each time. Denote TI as the transmission interval. Note that TI is the same for all LoRa devices.

**Figure 2:**
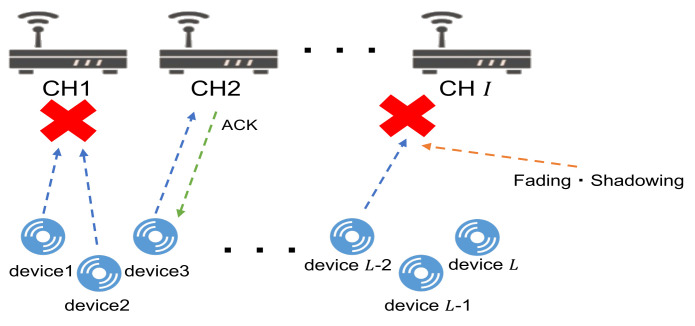
Channel access model.

The process of packet transmission in the LoRa system is summarized as follows. The transmission parameters, including the SF and channel, are first selected by the LoRa devices using the distributed MAB-based reinforcement learning methods implemented on them. After determining the transmission parameters based on the implemented learning methods, carrier sensing is performed to check the availability of the selected channel. If the selected channel is available, the LoRa device sends a packet to the GW using that channel. The feedback ACKnowledgement (ACK) or NACK information from the GW will be received at the LoRa devices’ side for a while after packet transmission, which is used to update the MAB-based reinforcement learning methods. If the ACK information is received, it represents that no packet collision or a capture effect occurred, and the packet from the LoRa device was successfully transmitted, as shown in the middle of Figure 2. On the other hand, the packet transmission fails for some reason if the NACK information is received. The reasons that cause the packet transmission failure may include that other LoRa devices transmit packets using the same channel and SF at the same time, as shown in the left side of Figure 2, causing packet collisions among them. In addition, the reason may include the interference from other IoT devices, or the signal is attenuated by shadowing due to a low SF value, resulting in an SNR value that is smaller than the threshold value that can be received, as shown in the right side of Figure 2.

The FSR was used to evaluate the performance of the MAB-based joint SF- and channel-selection methods in this paper. The FSR in the LoRa system at the *t*-th decision is defined as the ratio of the number of successful transmissions to the total number of transmission attempts, which is expressed as:

(1) $$FSR\left(t\right)=\frac{\sum_{l=1}^{L}r_{l}\left(t\right)}{\sum_{l=1}^{L}n_{l}\left(t\right)},$$

where $n_{l}\left(t\right)$ is the number of transmission attempts by device *l* and $r_{l}\left(t\right)$ is the number of successful transmissions at the time *t*. This paper aimed to maximize the FSR by the MAB-based decentralized learning methods, thereby improving the scalability of the overall LoRa application. The FSR maximization problem can be formulated as follows:

(2) $$\left(P\right)=max\sum_{t=1}^{T}FSR\left(t\right).$$

To achieve this goal, an appropriate SF must be selected based on the distance from the GW and the surrounding environment. Meanwhile, a channel less affected by other LoRa devices must be well chosen. In the LoRa system, packet collisions occur when they are transmitted on the same channel and SF at the same time. Hence, the SF and channel must be co-selected, and their relationship must be jointly considered in the selection.

## 4. Channel and SF Selection Based on MAB Algorithms

As mentioned in the previous section, LoRa devices must select appropriate parameters, such as the SF and channel, according to the communication environments. To achieve this goal, the SF–channel-selection problem was formulated as the MAB problem in this paper and solved by the MAB-based algorithms. In this section, we first introduce the relationship between the SF–channel-selection problem and the MAB problem. Next, the SF–channel-selection problem is formulated as two MAB problems with different structures, i.e., a combinatorial MAB-based and an independent MAB-based channel–SF-selection problems. Finally, we present the MAB algorithm for solving the formulated SF–channel-selection problems.

### 4.1. MAB and Channel–SF-Selection Problems

The MAB problem or bandit problem is one of the general problems first discussed by Robbins in [35]. In the MAB problem, the player selects a slot machine to play among several slot machines, aiming to maximize the number of coins he/she can earn by repeatedly playing [36]. The player needs to learn the probability of the number of coins for each slot machine to find the slot machine that pays the most by repeatedly playing. In other words, we have to perform exploration to gather information by playing slot machines other than the one with the best probability. On the other hand, if we perform more exploration than necessary, we cannot maximize the number of coins we can win. Hence, if we can estimate a good slot machine, we must play that slot machine to maximize the reward. The MAB problem is a decision-making problem that considers the trade-off between “exploration” for searching for a good slot machine and “exploitation” for playing a good slot machine to increase the coins in a series of trials.

In the most-straightforward formulation, the bandit problem has *K* slot machines with probability distributions ($D_{1}$,...$D_{K}$). The mean and variance of each probability distribution can be expressed as ($\mu_{1}$,…$\mu_{K}$) and ($\sigma_{1}$,…$\sigma_{K}$), respectively. The player aims to find the probability distribution with the largest expected value and tries to obtain as many rewards as possible in a sequence of trials. At each trial *t*, the player selects a slot machine m(t) and wins r(t) as a reward (i.e., r(t) coins). The bandit algorithm for solving the bandit problem can be described as a decision-making strategy determining the slot machine m(t) to be selected for each trial. Reward maximization is the most-used metric for evaluating the performance of a bandit algorithm. The bandit algorithm for solving the bandit problem will be described later in this section. The reward maximization problem can be expressed as follows, where *T* is the total number of trials.

(3) $$R_{T}=max\sum_{t=1}^{T}r\left(t\right).$$

As described in Section 2, we aimed to maximize the cumulative FSR by letting each device autonomously select the appropriate channel and SF using the ACK/NACK information. The problem of learning appropriate channels and SFs using only the ACK/NACK information can be transformed into the MAB problem: an IoT device (i.e., the player in the MAB problem) has *S* SFs and *I* channels (i.e., the slot machines in the MAB problem). The objective is to maximize the cumulative FSR (i.e., the cumulative rewards in the MAB problem). The relationship between the channel–SF-selection and the MAB problems is summarized in Table 3.

### 4.2. MAB-Based Channel–SF-Selection Problem

When the parameters to be selected are only SFs or only channels, i.e., when there is only one parameter to be selected, the MAB problem can be applied directly, as described in the previous subsection. However, to perform autonomous decentralized joint optimization of the channel- and SF-selection problem, we need to design the structure of the MAB-based channel–SF-selection method. In this subsection, we introduce two structures of the MAB-based channel–SF-selection problem, i.e., combinatorial and independent MAB-based channel–SF-selection problems.

#### 4.2.1. Combinatorial MAB-Based Channel–SF-Selection Problem

**Figure 3:**
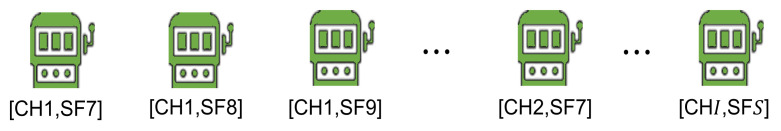
Combinatorial MAB-based channel–SF-selection problem.

We first describe the combinatorial MAB-based channel–SF-selection problem. In this problem, any combination of the SF and CH is configured as one slot machine, as shown in Figure 3. Hence, the number of slot machines is I×S. The best slot machine among these combinations is selected using the MAB algorithms by maximizing the reward (i.e., the FSR). The main design idea of this structure is that it is necessary to optimize the channel and SF considering their potential relationship since packets sent using the same channel and SF simultaneously will cause collisions in the LoRa system.

The combinatorial MAB-based channel–SF-selection problem process can be summarized as follows. The channel–SF is first selected based on the strategy of the MAB algorithms implemented on each LoRa device. Then, packets are sent using the selected SF and channel. The reward of the selected SF–channel combination is evaluated depending on whether the packet was successfully sent. For the next packet transmission time, each LoRa device dynamically selects the optimal SF–channel combination based on the updated evaluation and repeats this process until the time limit *T* is reached. The details of the combinatorial MAB-based channel–SF-selection problem are summarized in Algorithm 1.
**Algorithm 1** Combinatorial MAB-based channel–SF selection.1:Initialize the parameters used in each MAB algorithm2:**while** time t≤ *T* **do**3:    Select channel–SF set based on the MAB algorithm.4:    Send a packet using the selected SF and channel.5:    **if** the packet is transmitted, and the ACK frame is received **then**6:        Transmission successful.7:    **else**8:        Transmission failure.9:    **end if**10:   Update the corresponding parameters according to each MAB algorithm.11:   t=t+112:   Sleep for transmission interval TI.13:**end while**

#### 4.2.2. Independent MAB-Based Channel–SF-Selection Problem

**Figure 4:**
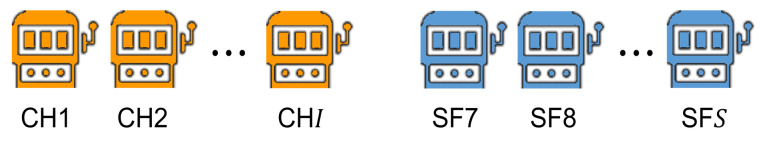
Independent MAB-based channel–SF-selection problem.

In the independent MAB-based channel–SF-selection structure, the channels and SFs are selected independently, aiming to optimize the channel and SF parameters, respectively. Two groups of machines are prepared; one group is used for SF selection, and the other group is used for channel selection. Hence, the numbers of the two types of machines are *S* and *I*, respectively. The number of machines for the independent MAB-based channel–SF-selection structure is S+I. Compared to the combinatorial MAB-based channel–SF-selection problem, the number of machines can be reduced to a great extent. By this, the memory requirements can be reduced. In addition, the efficiency of the search for the appropriate channel or SF may be increased. The schematic diagram of this structure is shown in Figure 4.

In the independent MAB-based channel–SF-selection problem, the SF is first selected based on the MAB algorithm implemented on the LoRa device. Similarly, a channel is selected. A packet is then sent using the chosen independent SF and channel. After that, the parameters related to the MAB algorithms are updated based on whether the packet was successfully transmitted. The process is repeated until the time limit *T*. The independent MAB-based channel–SF-selection problem’s details are shown in Algorithm 2. The computational complexity of the independent MAB-based channel–SF method is O(1), which was analyzed in our previous work [19].
**Algorithm 2** Independent MAB-based channel–SF-selection problem.1:Initialize the parameters of the MAB algorithm used for the SF and channel selection2:**while** time t≤ *T* **do**3:    Select the SF among the SF slot machines using the MAB algorithm.4:    Select the channel among the channel slot machines using the MAB algorithm.5:    Send a packet using the selected SF and channel.6:    **if** the packet is transmitted, and the ACK frame is received **then**7:        Transmission successful.8:    **else**9:        Transmission failure.10:  **end if**11:  Update the corresponding parameters for SF selection according to the policy of the MAB algorithm.12:  Update the corresponding parameters for channel selection according to the policy of the MAB algorithm.13:  t=t+114:  Sleep for transmission interval TI.15:**end while**

### 4.3. MAB Algorithms

In this paper, we focused on three MAB algorithms for solving the channel–SF-selection problem, that is the ε-greedy, UCB1, and ToW dynamics algorithms. In the following subsection, we discuss these three MAB algorithms in detail.

#### 4.3.1. ε-Greedy Algorithm

The ε-greedy algorithm is widely used for solving MAB problems because of its simplicity. In each trial, the slot machine with the highest reward probability determined by experience is selected and played with a probability of 1 −ε. On the other hand, the slot machines are randomly selected and played with probability ε. The policy of the ε-greedy algorithm is expressed below.

(4) $$p_{k_{ij}}\left(t\right)=\frac{R_{k_{ij}}\left(t\right)}{N_{k_{ij}}\left(t\right)},$$

(5) $$k_{ij}^{*}=\left\{\begin{array}{cc} {\arg\max}_{k_{ij}\in k_{j}}p_{k_{ij}}\left(t\right) & \mathrm{if}\;1-\epsilon ,\\ \mathrm{Randomly}\mathrm{selected} & \mathrm{otherwise}, \end{array}\right.$$

where *j* is the indicator of the channel and SF selection and j∈1,2,3, that is j=1 corresponds to the joint channel and SF selection in the combinatorial MAB-based channel–SF-selection problem and j=2 and j=3 correspond to the channel and SF selections in the independent MAB-based channel–SF-selection problem. $K_{j}$ is the number of arms corresponding to the structure *j*. $K_{1}$ is the number of SF and channel combinations for the combinatorial MAB-based channel–SF selection. The value of $K_{1}$ is I×S. The values of $K_{2}$ and $K_{3}$ are equal to the number of SFs *S* and channels *I*, respectively, for the independent MAB-based channel–SF-selection problem. $k_{j}$ is the set of slot machines. $k_{1}=${$s_{1}i_{1},s_{1}i_{2},\cdots ,s_{1}i_{I},s_{2}i_{1},\cdots ,s_{S}i_{I}\}$, i.e., the set of channel and SF combinations for the combinatorial MAB-based channel–SF-selection problem. $k_{2}=${$s_{1},s_{2},\cdots ,s_{S}\}$ and $k_{3}=${$i_{1},i_{2},\cdots ,i_{I}\}$, i.e., the set of channels and that of SFs for the independent MAB-based channel–SF-selection problem. $k_{ij}$ is the *i*-th slot machine in $k_{j}$. $N_{k_{ij}}$ is the number of times the arm $k_{ij}$ is selected at iteration *t*. $R_{k_{ij}}$ is the number of successful transmissions among $N_{k_{ij}}$, i.e., the number of times the ACK information is received.

#### 4.3.2. Upper Confidence Bound1

Upper Confidence Bound (UCB) algorithm sequences were proposed by Auer and Bianchi in [37]. The UCB1 algorithm is the simplest one among the UCB series. The UCB1 algorithm selects the slot machine based on the average reward and the number of times each slot machine is played. This algorithm considers the upper bound of the confidence interval. The slot machine $X_{k_{ij}}\left(t\right)$ is selected in the *t*-th trial after playing each slot machine once, according to the following equation.

(6) $$X_{k_{ij}}\left(t\right)=\frac{R_{k_{ij}}\left(t\right)}{N_{k_{ij}}\left(t\right)}+\sqrt{\frac{2\ln t}{N_{k_{ij}}\left(t\right)}},$$

(7) $$k_{ij}^{*}={\arg\max}_{k_{ij}\in k_{j}}X_{k_{ij}}\left(t\right).$$

Auer et al. also proposed a UCB1-Tuned algorithm, which considers not only the empirical mean value of each slot machine, but also the empirical variance [37]. This algorithm is the best-performing algorithm among the current MAB algorithms. In the UCB1-Tuned algorithm, the slot machine is selected based on the following equation in each trial.

(8) $$X_{k_{ij}}\left(t\right)=\frac{R_{k_{ij}}\left(t\right)}{N_{k_{ij}}\left(t\right)}+\sqrt{\frac{\ln t}{N_{k_{ij}}\left(t\right)}\min\left(1/4,V_{k_{ij}}\left(t\right)\right)},$$

(9) $$k_{ij}^{*}={\arg\max}_{k_{ij}\in k_{j}}X_{k_{ij}}\left(t\right).$$

where $V_{k_{ij}}\left(t\right)$ is based on the estimated variance, which can be expressed as follows. $\sigma_{k_{ij}}$ in the equation is the variance of the obtained reward.

(10) $$V_{k_{ij}}\left(t\right)=\sigma_{k_{ij}}^{2}+\sqrt{\frac{2\ln t}{N_{k_{ij}}\left(t\right)}}.$$

#### 4.3.3. Tug of War Dynamics

**Figure 5:**
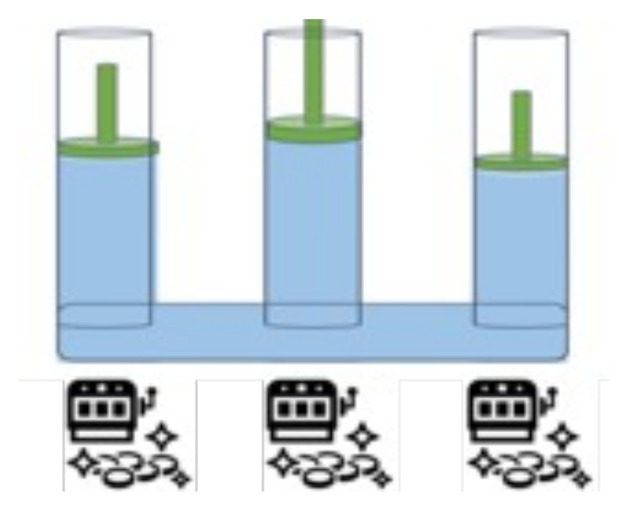
ToW dynamics.

The ToW is a simple method with low computational complexity. It has been analytically validated that the ToW dynamics is efficient in maximizing stochastic rewards under dynamic environments where the reward probabilities of the arms change frequently [38,39,40,41]. The essential element of the ToW dynamics is a volume-conserving physical object. It assumes that each slot machine is allocated to multiple cylinders with branches filled with an incompressible fluid, as shown in Figure 5. The volume is then updated by pushing and pulling the corresponding cylinders depending on whether the slot machine is rewarded for a trial at time *t*. In addition, since the cylinders are connected, as shown in the figure, a volume increase in one part is immediately compensated by a volume decrease in another part. In the ToW dynamics, the arm $k_{ij}^{*}$ with the height cylinder interface value $X_{k_{ij}}$ is selected. The following formula expresses $X_{k_{ij}}$.

(11) $$X_{k_{ij}}\left(t\right)=Q_{k_{ij}}\left(t-1\right)-\frac{1}{K_{j}-1}\sum_{k_{ij}^{\prime }\neq k_{ij}}^{K_{j}}Q_{k_{ij}^{\prime }}\left(t\right)+{\mathrm{osc}}_{k_{ij}}\left(t\right).$$

There are various possibilities for adding oscillations ${osc}_{k_{ij}}\left(t\right)$. References [41,42] studied the impact of oscillations on the efficiency of decision-making in detail, which is beyond the scope of this paper. In this paper, the incompressible liquids oscillate autonomously according to the following equation.

(12) $${\mathrm{osc}}_{k_{ij}}\left(t\right)=A\cos\left(\frac{2\pi t}{K_{j}}+\frac{2(k_{ij}-1)\pi }{K_{j}}\right).$$

In addition, $Q_{k_{ij}}\left(t\right)$ is the estimated compensation for each arm, which is derived by the following equation:

(13) $$Q_{k_{ij}}\left(t\right)=\left\{\begin{array}{cc} \alpha Q_{k_{ij}}\left(t-1\right)+\Delta Q_{k_{ij}}\left(t\right) & \mathrm{if}k_{ij}=k_{ij}^{*},\\ \alpha Q_{k_{ij}}\left(t-1\right) & \mathrm{otherwise}, \end{array}\right.$$

where α (0 < α < 1) is the discount factor for estimated compensation. By introducing α, we can control the impact of the past learning experience on the present to adapt to natural communication environments where the channel conditions may change dynamically. $\Delta Q_{k_{ij}}\left(t\right)$ is given by the following formula.

(14) $$\Delta Q_{k_{ij}}\left(t\right)=\left\{\begin{array}{cc} +1 & \mathrm{if}\mathrm{rewarded},\\ -\omega_{ij}\left(t\right) & \mathrm{otherwise}. \end{array}\right.$$

In other words, if the transmission is successful and the ACK is received, the Q value of the selected arm (parameter) gains “+1” as a reward. By this, the height of the fluid interface for that arm is increased. Conversely, when the transmission fails and an ACK is not received, the corresponding arm (parameter) is updated with the punishment $-\omega_{ij}\left(t\right)$. By this, the interface value of the selected arm is decreased. Correspondingly, the interface value of other arms increases. Here, $\omega_{ij}\left(t\right)$ is expressed as:

(15) $$\omega_{ij}\left(t\right)=\frac{p_{ij}{}_{1\mathrm{st}}\left(t\right)+p_{ij}{}_{2\mathrm{nd}}\left(t\right)}{2-(p_{ij}{}_{1\mathrm{st}}\left(t\right)+p_{ij}{}_{2\mathrm{nd}}\left(t\right))}.$$

where $p_{ij1\mathrm{st}}\left(t\right)$ and $p_{ij2\mathrm{nd}}\left(t\right)$ are the arms with the highest and second-highest reward probabilities among all arms at time *t*, respectively. The reward probability is given by Equation (4). In the ToW dynamics, $N_{k_{ij}}\left(t\right)$ is the number of times the arm $k_{ij}$ is selected by time *t* and $R_{k_{ij}}\left(t\right)$ is the number of successful transmissions using the arm $k_{ij}$ by time *t*. $N_{k_{ij}}\left(t\right)$ and $R_{k_{ij}}\left(t\right)$ are given by the following equations, respectively.

(16) $$N_{k_{ij}}\left(t\right)=\left\{\begin{array}{cc} 1+\beta N_{k_{ij}}\left(t-1\right) & \mathrm{if}k_{ij}=k_{ij}^{*},\\ \beta N_{k_{ij}}\left(t-1\right) & \mathrm{otherwise}, \end{array}\right.$$

(17) $$R_{k_{ij}}\left(t\right)=\left\{\begin{array}{cc} 1+\beta R_{k_{ij}}\left(t-1\right) & \mathrm{if}k_{ij}=k_{ij}^{*}\;\mathrm{and}\;\mathrm{rewarded},\\ \beta R_{k_{ij}}\left(t-1\right) & \mathrm{otherwise}, \end{array}\right.$$

where β (0 < β≤ 1) is the forgetting rate.

## 5. Implementation and Performance Evaluation of the MAB-Based Channel–SF-Selection Methods

This section evaluates the proposed combinatorial MAB-based joint channel and SF-selection methods, including the ToW-dynamics-based, UCB1-based, ϵ-greedy-based methods, and random methods by conducting experiments using actual LoRa devices. Specifically, the FSR under different numbers of LoRa devices and the rate of the SF selection for the LoRa devices at different positions when only selecting SF were evaluated first. Then, the performance of the FSR and FI when selecting both the SF and channel was evaluated exhaustively. This section describes the experiment settings first, followed by the performance evaluation when only selecting the SF and selecting both the SF and channel, respectively.

### 5.1. Experiment Settings

**Figure 6:**
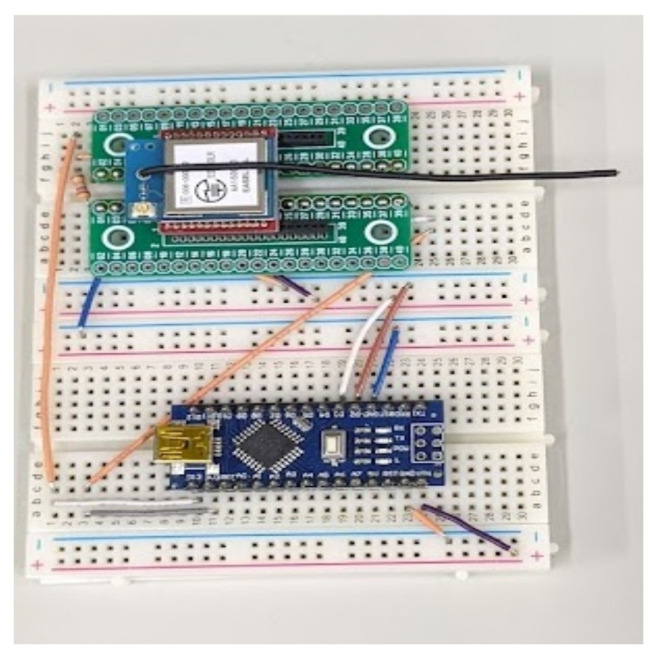
LoRa device.

**Figure 7:**
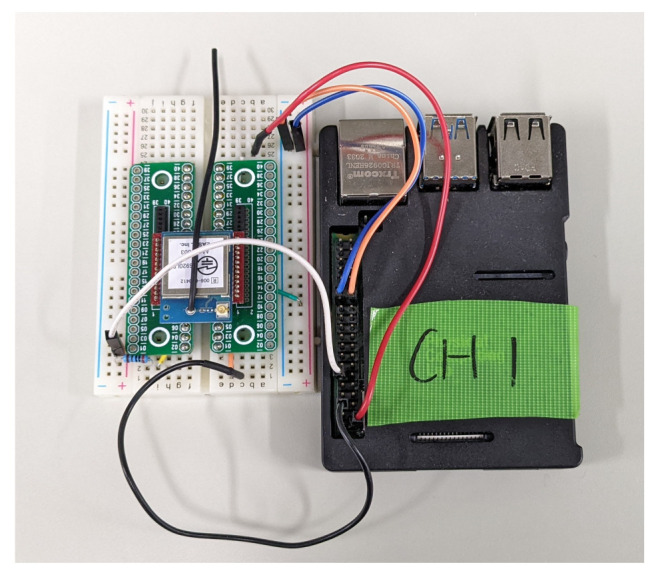
GW.

**Figure 8:**
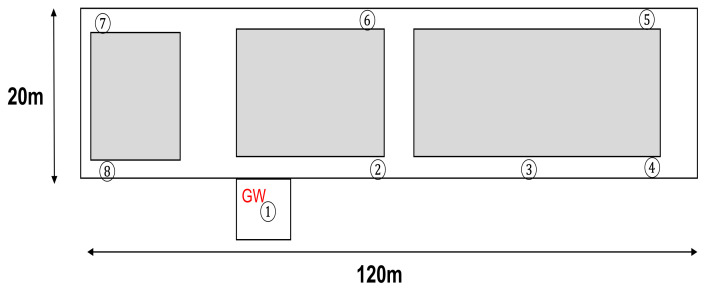
Experiment field.

The MAB-based SF–channel-selection methods use a LoRa module ESP320LR that supports LoRa communication in the 920 MHz band. A Raspberry Pi and a battery-powered Arduino mini pro were used as the GW and LoRa device controllers, respectively. The component parts of the LoRa device and the GW are shown in Figure 6 and Figure 7, respectively. The communication between the GW and LoRa devices used a LoRa wireless link. The GW was connected to a common network server over a standard IP protocol stack using a WiFi router. Using the implemented LoRa system, we evaluated the performance of the (i) ϵ-greedy-based, (ii) UCB1-based, (iii) ToW-dynamics-based joint channel and SF selection in the combinatorial and independent MAB-based channel–SF-selection methods, and (iv) the random channel–SF selection. Among the compared methods, the UCB1-based method was introduced in [27,32], while the MAB-based independent methods were proposed in [19]. By comparing the recently published results, we aimed to justify that the proposed ToW-dynamics-based combinatorial transmission selection method has advanced the state-of-the-art in this field. In the experiments, the impact of the transmission intervals, the number of LoRa devices, and the locations of the LoRa devices on the communication quality, especially on the FSR, was evaluated. The experiments were conducted indoors in a 120 m × 20 m rectangular area on the fifth floor of a concrete wall building. The LoRa devices were placed in several locations. The diagram of the experiment field is shown in Figure 8. As shown in Figure 8, the GW was deployed in Position ➀ of a room termed Room 1 in this paper. The LoRa devices in Position ➀ were deployed in the same room as the GW, i.e., Room 1, and there was no obstacle between them, guaranteeing a Line of Sight (LoS) path. Meanwhile, except for the LoRa devices deployed at Position ➀ in Room 1, all other LoRa devices were deployed in the corridor, i.e., Positions ➁∼➇ shown in Figure 8. The gray parts in Figure 8 are the other rooms with walls except Room 1, resulting in a Non-Line-of-Sight (NLOS) path with the GW. The Received Signal Strength Indicator (RSSI) from each location is summarized in Table 4. The LoRa devices deployed in the positions with a lower RSSI should select a higher SF due to the lower received strength at the GW. We considered two scenarios to evaluate the impact of the channel and SF selections on the network performance. The first scenario was where the LoRa device only performed SF selection. To verify the effectiveness of the MAB-based methods, we evaluated the performance of the FSR of the MAB-based methods compared with the selection randomly. Moreover, to confirm that the SFs needed to be selected appropriately according to the reception strength from each location, we evaluated the selection rate of the SFs at different positions. In the second scenario, both channel–SF selections were performed using the combinatorial and independent MAB-based selection methods described in the previous section. In this scenario, we first evaluated the effect of the structure of the MAB-based methods on the FSR. Then, we evaluated the performance of the FSR with varying numbers of LoRa devices and transmission intervals for the combinatorial MAB-based selection methods in detail. Note that the results shown below were the average value over 10 repetitions of the experiment in each setting.

### 5.2. Performance Evaluation of the SF Selection

In this subsection, we describe the experimental results in the setting where the LoRa device only performed SF selection. The effectiveness of the distributed approaches using the MAB algorithm was first evaluated. Then, the impact of the SF selection on the distance from the GW was evaluated based on the ToW-dynamics-based SF-selection method. In the experiments, the LoRa devices were placed at three locations, i.e., Positions ➀, ➁, and ➂, in the experimental field shown in Figure 8. The number of channels was set as one, while the channel used in the experiments was CH1 working at 920.6 MHz band. The transmission interval was set to 20 s. Packets with a payload of 50 bytes were sent in each data transmission. The parameter ε in the ε-greedy method was set as 0.1, and the forgetting parameters α and β in the ToW dynamics were set as 0.9. The parameters related to the experiments are listed in Table 5.

**Figure 9:**
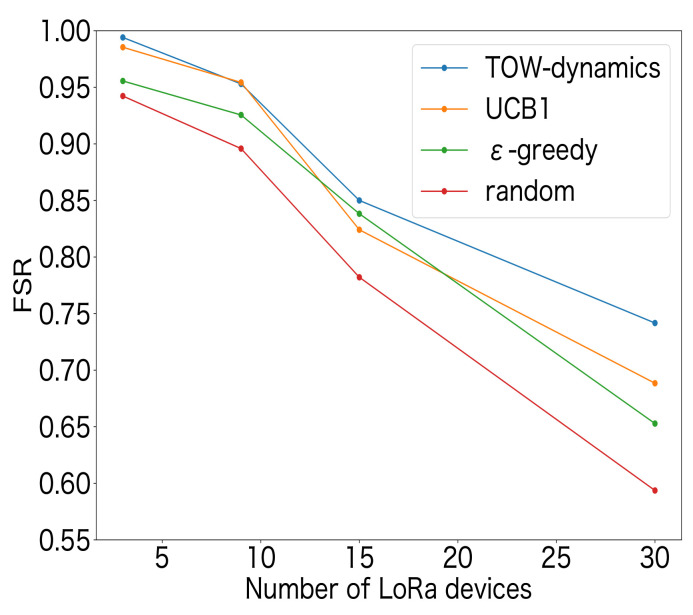
FSR for SF selection.

**Figure 10:**
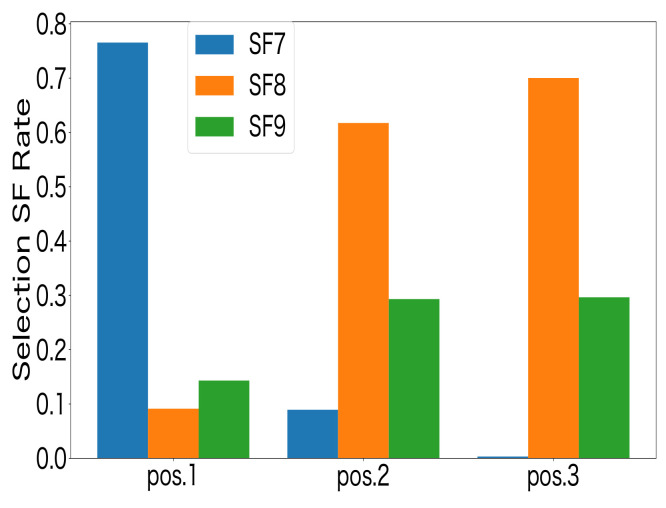
SF selection using ToW dynamics.

Figure 9 shows the performance in terms of the FSR for the MAB-based SF-selection methods and the random method. The number of LoRa devices was set to 3, 9, 15, and 30. The LoRa devices were deployed at each location equally, i.e., 1 device was deployed at each location when the total number of LoRa devices was 3, and 10 devices were deployed at each location when the total number of LoRa devices was 30. From Figure 9, it can be seen that the FSR decreased as the number of LoRa devices increased for all approaches. This was due to the increase in packet collisions as the number of LoRa devices increased. In addition, the MAB-based SF-selection methods can achieve a higher FSR than the random-based selection method, indicating that the distributed reinforcement learning approaches were effective. Moreover, compared to the existing studies on decentralized parameter selection using UCB1 in [27,32] and ϵ-greedy in [3], the ToW dynamics algorithm can achieve a higher FSR. A comparison of the FSR values showed that, as the number of LoRa devices increased, the difference between the ToW and the other MAB-based methods increased, which indicated that the ToW algorithm is more suitable for large-scale LoRa systems. Figure 10 shows the ratio of the SF selection at each position for the ToW-dynamics-based SF-selection method. The number of LoRa devices was set to 30 in the experiments. The results showed that a higher SF was selected by the LoRa devices farther from the GW. The reason was that the reception strength at the GW was weak for farther LoRa devices. A higher SF that could resist the noise was selected to guarantee successful transmission at a more-distant location with a lower receiver sensitivity and SNR threshold. In summary, the ToW-dynamics-based SF selection can select the appropriate SF for the LoRa devices deployed in different positions without prior information.

### 5.3. Performance Evaluation of the Channel–SF Selection

This subsection introduces the experimental results for joint channel and SF selection using the MAB-based methods described in the last section. We first evaluated the impact of the different MAB structures on the performance of the FSR and the FI. Then, we evaluated the impact of the number of users and the transmission interval on the FSR for the combinational MAB-based method. Finally, we evaluated the effect of the position of the LoRa devices deployed on the performance of the FSR.

#### 5.3.1. Combinatorial MAB-Based Problem vs. Independent MAB-Based Problem

The performances of the FSR and FI for the two structures of the MAB-based channel–SF-selection methods were evaluated. In the experiments, the number of LoRa devices was set as 30, and 10 devices were deployed at Positions ➀, ➁, and ➂, respectively. Three channels were used in the experiments, i.e., CH1, CH4, and CH7. The experimental parameter settings are shown in Table 6.

**Figure 11:**
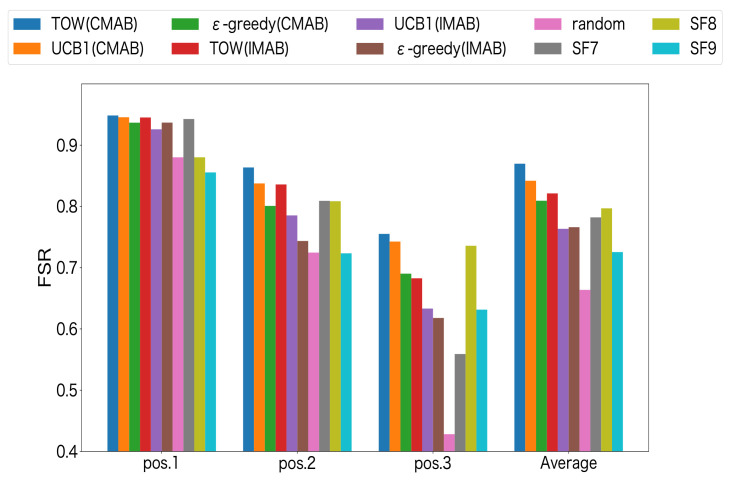
FSR for channel–SF selection.

**Figure 12:**
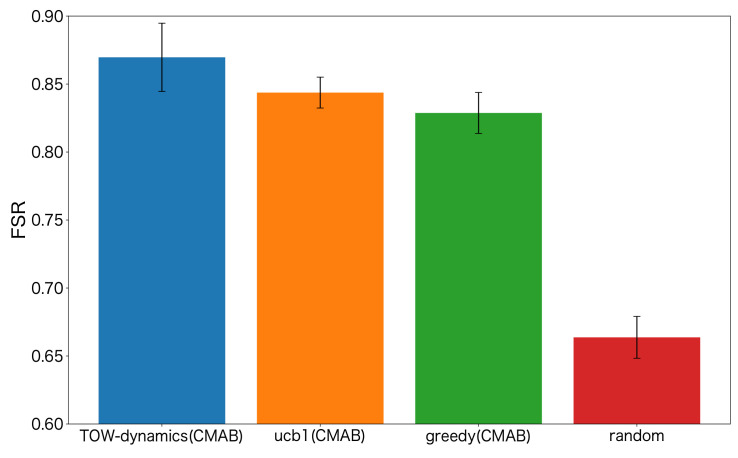
Confidence intervals for channel–SF selection.

Figure 11 shows the FSR for different methods at each location. CMAB and IMAB denote the combinatorial and independent MAB-based channel–SF methods, respectively. SF7-SF9 denotes the results with all LoRa devices fixed to the same SF and the channels equally allocated to 10 devices on each of the three channels. The experimental results showed that the FSR decreased with the distance between LoRa devices and the GW increasing. The reason may be that the time on air for the LoRa devices that were farther from the GW was longer, which may cause collisions with high probability. Moreover, the combinatorial MAB-based methods could achieve a higher FSR than independent MAB-based methods for all the MAB algorithms. Since packet collisions in LoRa systems occur when both the channel and SF are the same, the combinatorial MAB-based method, which can account for their relationship, showed better results. The lowest FSR was obtained among the fixed allocation methods when the SFs of all LoRa devices was set to 9 for average.This was because the packet time on air was longer when the SF is set to 9, which increased the probability of packet collisions. On the other hand, the highest FSR among the fixed methods was achieved when the SF was set to 8 for average, but even at this FSR, the success rate was still lower than the MAB algorithm based on the CMABstructure. Furthermore, since the fixed method allocates channels equally based on prior knowledge of the number of LoRa devices, the MAB-based method was more effective in the actual network, where prior information was unavailable. Figure 12 shows the confidence interval of the FSR for combinational MAB-based and random channel–SF selection methods. From this, we can see that the combinational ToW-based channel–SF-selection method could achieve the highest FSR compared to the other methods.

**Figure 13:**
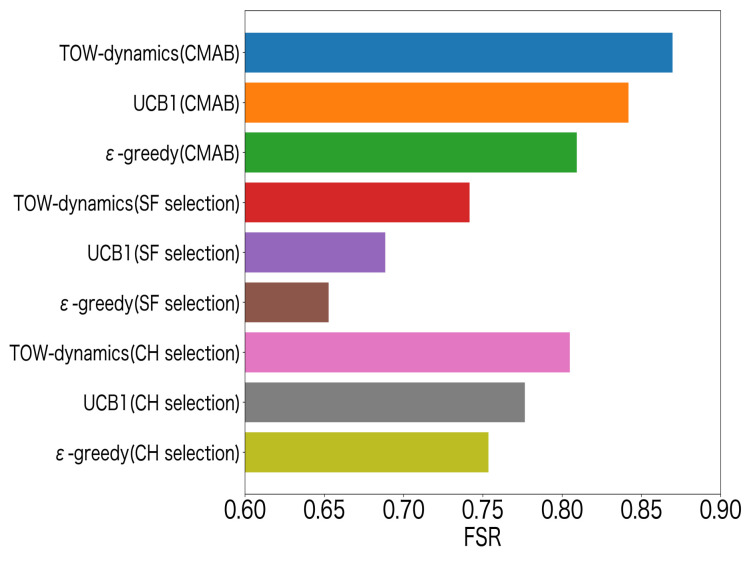
FSR for channel–SF selection, channel selection, and SF selection.

**Figure 14:**
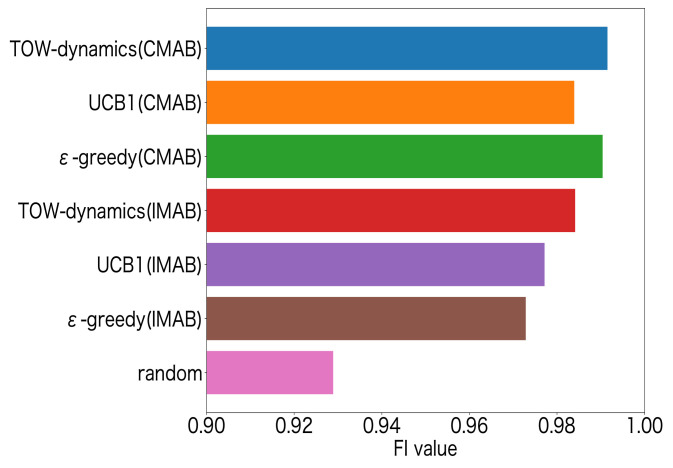
FI for channel–SF selection.

In Figure 13, we compare the FSR of all the MAB algorithm methods with the CMAB structure (CH–SF selection), the CH selection by all the MAB algorithms, and the SF selection by all the MAB algorithms. Among them, all the MAB algorithms methods with the CMAB structure are our proposed method, and the CH or SF selection by all the MAB algorithms represents the existing methods. From Figure 13, we can see that the ToW dynamics method with the CMAB structure was superior in the FSR to other methods. Figure 14 shows the performance of the FI for the MAB-based channel–SF-selection methods. The FI was used to evaluate the fairness of the LoRa devices that were deployed at Positions ➀–➂, which is expressed by the following equation:

(18) $$\mathrm{FI}=\frac{{\left(\sum_{l=1}^{L}FSR_{l}\right)}^{2}}{L\sum_{l=1}^{L}{\left(FSR_{l}\right)}^{2}}.$$

From the results, we can see that the MAB-based selection methods achieved a much higher FI compared to the random-based selection method, which verified the effectiveness of the MAB-based methods. Moreover, similar to the FSR evaluation, the combinatorial MAB-based channel–SF selection also could achieve a higher FI compared to the independent MAB-based methods. This was due to the high FSR in LoRa devices far from the GW, such as Positions ➁ and ➂, in the combinatorial MAB-based method.

#### 5.3.2. Effect of the Experimental Parameters on FSR for CMAB Methods

To measure the effectiveness of the experimental parameters on the FSR, the experiments were performed by varying the number of LoRa devices and the transmission interval. The number of LoRa devices was set to 3, 9, 15, and 30, while the transmission intervals were set to 20 s and 50 s. The structure of the method used in the experiments was a combinational MAB-based channel–SF-selection method since its superiority was shown in our previous experiments. The other parameters related to the experiments are listed in Table 7.

**Figure 15:**
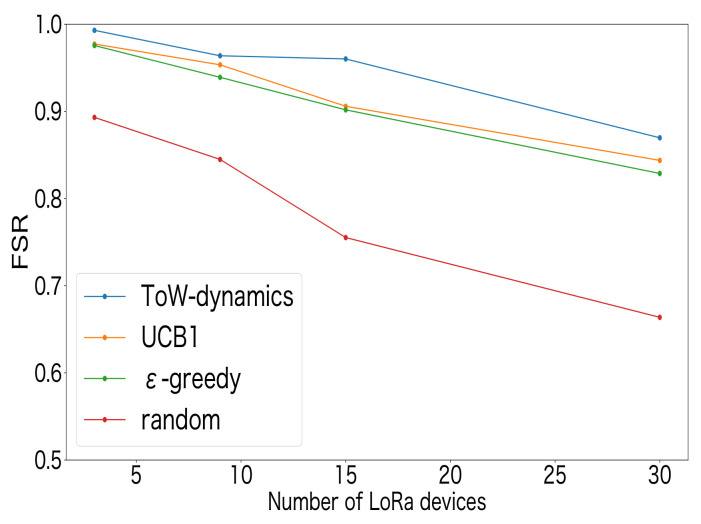
FSR vs. the number of LoRa devices.

We first evaluated the effect of the number of LoRa devices on the FSR. In the experiments, the transmission interval was set as 20 s. Figure 15 shows the results, from which we can see that the FSR decreased with the increase of the number of LoRa devices. The reason was that an increase in the number of LoRa devices increased the number of transmitted packets, increasing collisions and interference. In addition, compared to the experimental results shown in Figure 9, where only the SF selection was considered, the effect of the number of available channels on the FSR was small when the number of LoRa devices was small. However, when the number of LoRa devices increased, Figure 15, where multiple channels were used, shows a better FSR. This indicated the necessity of selecting both the SF and channel.

**Figure 16:**
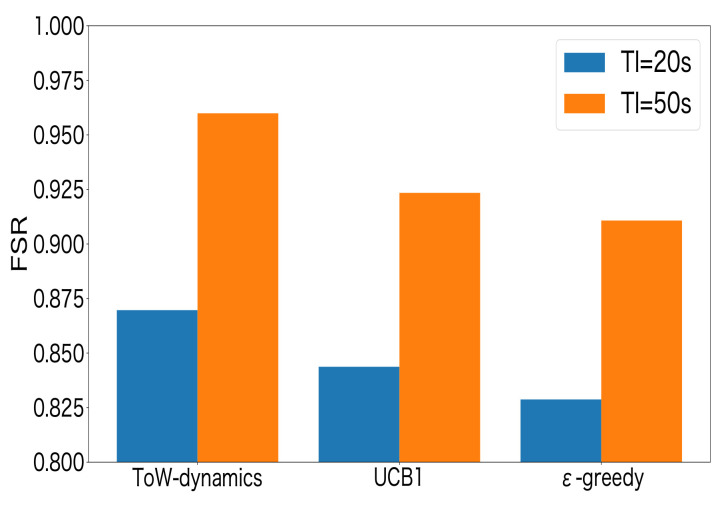
FSR vs. the transmission interval.

Then, we evaluated the effect of the transmission interval on the FSR. In the experiments, the transmission intervals were set to 20 s and 50 s. The number of LoRa devices was set to 30. Figure 16 shows the experimental results. From the results, it can be seen that a higher FSR could be achieved for all of the combinational MAB-based channel–SF-selection methods when the transmission interval was 50 s compared to the case where the transmission interval was 20 s. This indicated that the transmission interval should be set appropriately according to the requirements of the LoRa applications. The FSR may increase by adjusting the transmission interval autonomously, which will be studied in our future work.

#### 5.3.3. Effect of the Setting Position of LoRa Devices on FSR

**Figure 17:**
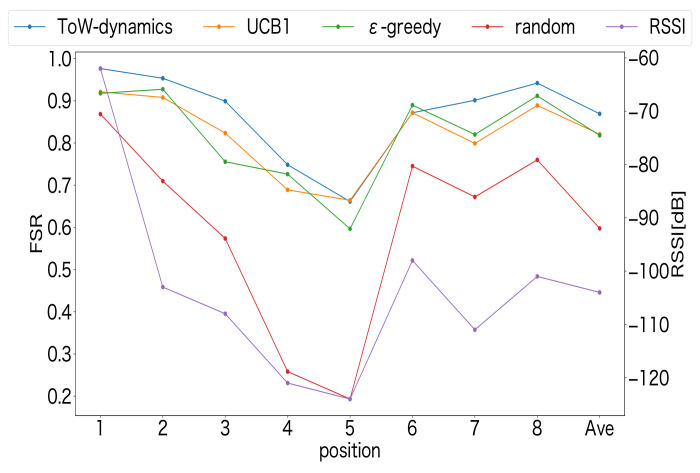
FSR at Positions ➀–➇.

In our previous experiment, the LoRa devices were deployed at Positions ➀–➂. In the following experiments, the LoRa devices were deployed at Positions ➀–➇. The number of LoRa devices was set to 24. At each position, three LoRa devices were deployed. The other parameters used in the experiment were same as shown in Table 7, and the TI was 20 s. Figure 17 shows the FSR and the average RSSI of the received packets at each position. Similar to the previous results, it can be seen that the MAB-based channel–SF-selection methods achieved a much higher FSR than the random method, regardless of the positions. In particular, the random method showed a significant decrease in the FSR at lower RSSI values, indicating the necessity of using the MAB algorithms to select the channel and SF appropriately depending on the deployed positions of the LoRa devices. In addition, the FSR at each position was proportional to the RSSI value for all methods. The reason may be that the larger SF was selected by the LoRa devices deployed in the positions with a lower RSSI, which increased the time on air of the transmitted packets. Hence, the probability of collisions may increase.

## 6. Conclusions

In this paper, we implemented and evaluated lightweight autonomous distributed reinforcement learning methods for joint channel and SF selection in a practical larger-scale LoRa system. As a result, we were able to verify the necessity of dynamically selecting both the SF and channel. Specifically, the results showed that the channel–SF selection using the MAB-based methods was effective compared to random selection, especially in situations where the LoRa devices were distributed in various locations. Specifically, when the difference between the FSR of the proposed ToW dynamics and that of random selection was highest, the achieved FSRs of the ToW dynamics and random selection were 0.86919 and 0.59761, respectively. Hence, the percentage difference of the achieved maximum FSRs for the ToW dynamics and random selection was 145%. Besides, the ToW-dynamics-based method outperformed other MAB-based methods, such as UCB1, used in the recently published results, whether with the combinational or independent structure. In addition, the structures of the MAB-based methods and the other communication parameters also greatly affected the FSR and FI. Specifically, the combinational MAB-based methods could achieve a higher FSR and FI than the independent MAB-based methods considered in our previous research. Hence, the relevance of the channel and SF is a very important factor for the communication performance of larger-scale LoRa systems. Moreover, the FSR can be improved by jointly selecting the channel and SF compared to only selecting the SF. Furthermore, by increasing the transmission interval, the FSR can be improved to a great extent. In our future work, we will consider the joint channel and SF selection in outdoor, longer-distance environments, the optimization of other communication parameters, and the energy efficiency of the MAB-based methods.

## Figures and Tables

**Table 1:** Communication parameters with different SFs in LoRa systems.

SF	Bit Rate (kbps)	Receiver Sensitivity (dBm)	SNR Threshold (dB)	FSR for Pure ALOHA
7	5.47	−123	−6	0.79514
8	3.13	−126	−9	0.67353
9	1.76	−129	−12	0.51529
10	0.98	−132	−15	0.61592
11	0.54	−133	−17.5	0.42236
12	0.29	−136	−20	0.20450

**Table 2:** The comparison of relevant schemes.

Reference	Centralized	Decentralized	SF	CH	Implementation
[15]	✓	✕	✓	✕	✕
[20]	✓	✕	✓	✕	✕
[21]	✓	✕	✓	✕	✕
[22]	✓	✕	✓	✕	✕
[23]	✓	✕	✓	✕	✕
[24]	✓	✕	✓	✕	✕
[25]	✓	✕	✓	✕	✕
[26]	✓	✕	✓	✕	✓
[27]	✓	✕	✓	✕	✕
[28]	✓	✕	✓	✕	✕
[18]	✓	✕	✕	✓	✕
[29]	✓	✕	✕	✓	✕
[30]	✓	✕	✓	✓	✕
[31]	✕	✓	✓	✕	✕
[32]	✕	✓	✓	✕	✕
[33]	✕	✓	✕	✓	✓
[34]	✕	✓	✕	✓	✓
[19]	✕	✓	✓	✓	✓

**Table 3:** Correspondence of the MAB and channel–SF-selection problems.

MAB Problem	Channel–SF-Selection Problem
Player	IoT device
Slot machine	SF/CH
Reward: coin	Reward: ACK/NACK information
Objective: maximize the total number of coins	Objective: maximize the FSR

**Table 4:** RSSI from each position.

Position	RSSI (dBm)
1	−62
2	−103
3	−108
4	−121
5	−124
6	−98
7	−111
8	−101

**Table 5:** Parameter settings for the experiments of SF selection.

Parameter	Value
Number of LoRa Devices *L*	3, 9, 15, 30
Location	➀, ➁, ➂
Bandwidth BW	125 kHz
Channel	CH1 (920.6 MHz)
SF	7, 8, 9
Transmission Interval TI	20 s
Payload Length *L*	50 bytes
Transmission Power TP	13 dBm
Time Limit *T*	200 times

**Table 6:** Parameter settings for channel–SF selection (CMAB vs. IMAB).

Parameter	Value
Number of LoRa Devices *L*	30
Location	➀, ➁, ➂
Bandwidth BW	125 kHz
Channel	CH1, CH4, CH7
SF	7, 8, 9
Transmission Interval TI	20 s
Payload Length *L*	50 bytes
Transmission Power TP	13 dBm
Time Limit *T*	200 times

**Table 7:** Parameter settings for channel–SF selection (CMAB).

Parameter	Value
Number of the LoRa Devices *L*	3, 9, 15, 30
Location	➀, ➁, ➂
Bandwidth BW	125 kHz
Channel	CH1, CH4, CH7
SF	7, 8, 9
Transmission Interval TI	20, 50 s
Payload Length *L*	50 bytes
Transmission Power TP	13 dBm

## Data Availability

The data that support the findings of this study are available from the corresponding author upon reasonable request.
